# Clinical Outcomes of Bipolar Hemiarthroplasty with a Conjoined Tendon-Preserving Posterior Approach for Femoral Neck Fractures

**DOI:** 10.3390/medicina60030356

**Published:** 2024-02-21

**Authors:** Hidetatsu Tanaka, Yu Mori, Atsushi Noro, Toshihisa Yano, Toshimi Aizawa, Keiji Masuda

**Affiliations:** 1 Department of Orthopaedic Surgery, Yamagata City Hospital Saiseikan, 3-26, Nanokamachi, Yamagata 990-8533, Japan; hidetatsu.tanaka@med.tohoku.ac.jp (H.T.);; 2 Department of Orthopaedic Surgery, Tohoku University Graduate School of Medicine, 1-1 Seiryo-machi, Aoba-ku, Sendai 980-8574, Japan; toshi-7@med.tohoku.ac.jp

**Keywords:** conjoined tendon-preserving posterior, bipolar hemiarthroplasty, femoral neck fracture, postoperative dislocation, elderly person, body mass index, complication

## Abstract

*Background and Objectives*: The conventional posterior approach in the lateral decubitus position is widely used for femoral neck fractures in femoral hemiarthroplasty. Postoperative dislocation is the major problem with this approach. The conjoined tendon-preserving posterior (CPP) approach is a less invasive surgical approach than the conventional posterior approach to the hip, maintains posterior stability, and preserves short external rotators and joint capsules. However, the mention was required to avoid muscle damage and whether muscle damage affects postoperative dislocation or not. The current study aimed to evaluate the clinical results of the CPP approach in hemiarthroplasty for femoral neck fractures and identify muscle damage risk factors. *Materials and Methods*: This study was a retrospective cohort study and included 170 hips in 168 patients. The mean age at the operation was 81.2 years. The preservation rate of the internal obturator muscle and gemellus inferior muscle and factors related to intraoperative short rotator muscle injury were investigated retrospectively. The postoperative complications and the relation between muscle damage and postoperative dislocation were investigated. *Results*: In the four hips (2.3%) with the obturator internus muscle damage, thirty-eight hips (22.4%) with gemellus inferior muscle damage were detected; in the muscle-damaged cases, the high body mass index (BMI) was significantly higher. The complication occurred in four hips (2.3%), including postoperative posterior dislocation in one hip without muscle damage (0.6%). Postoperative infection occurred in one hip (0.6%), and peroneal or sciatic nerve paralysis was suspected in two hips (1.1%). *Conclusions*: Compared to the conventional posterior approach in previous reports, the CPP approach reduces postoperative dislocation. A higher BMI is a risk factor for muscle damage, and the gemellus inferior muscle damage has no effect on postoperative dislocation. The CPP approach for BHA appeared to be an effective treatment method.

## 1. Introduction

The incidence of femoral neck fractures is increasing with the aging society in Japan [1]. Bipolar hemiarthroplasty (BHA) is a commonly performed procedure for treating displaced femoral neck fractures. Surgeons can use several approaches, like the posterior, anterior, or lateral approach, each presenting pros and cons. The posterior approach is one of the common surgical procedures for femoral neck fractures. Since hip fractures expose patients to the risk of reduced life expectancy and functional decline, the development of surgical procedures that can restore function more quickly is an important issue. Postoperative dislocation after BHA is a severe complication that compromises patients’ quality of life and mortality [2]. The dislocation rate with the posterior approach has been reported to be 3.8–13.0% [3,4,5,6,7,8], and the postoperative dislocation rate of the BHA with the posterior approach is higher than the anterior approach [7,9,10]. Several articles recommend against the use of the posterior approach because the risk of dislocation is eight times higher with the posterior approach than with the lateral approach, and the risk of recurrent dislocation is higher with the posterior approach [11,12]. The BHA through the posterior approach should be re-evaluated since it is associated with higher rates of postoperative dislocation than other approaches and provides no significant advantage [12]. The increased hip range of motion following surgery that can result from the lack of joint rigidity, and the co-occurrence of postoperative delirium and dementia in patients with femur neck fractures may raise the risk factor for dislocation [8]. Preserving soft tissue is important to reduce the risk of postoperative dislocation. Regarding contiguity, muscle atrophy, and dislocation, the piriformis tendon preservation strategy used during the posterior approach in total hip arthroplasty (THA) is said to be superior to the reattachment technique [13]. An external rotator preservation (ERP) procedure for THA described by Kim et al. preserves muscles from the piriformis to the internal obturator muscle and has good outcomes with no cases of postoperative dislocation [14].

The conjoined tendon-preserving posterior (CPP) approach in BHA for femoral neck fractures has been reported to address postoperative dislocation by Nakamura et al. [15]. The CPP approach is a modified conventional posterior approach, which preserves the piriformis muscles and the conjoined tendon composed of the superior gemellus, internal obturator, and inferior gemellus muscles, as well as the joint capsule covered by the conjoined tendon [15,16]. The minimally invasive external rotation preserving procedure, which preserves muscles ranging from the piriformis to the internal obturator, was used. However, postoperative dislocation occurred in their institute [14,15]. They thought it preserves the areas of the short external rotator muscles and capsular ligament through caudal expansion in order to achieve increased joint stability and prevent dislocation [15]. The posterior `wall` consisted of the posterior soft tissues, reducing the rate of postoperative dislocation compared to the other posterior approaches.

In our institute, the CPP approach has been used since April 2018 to reduce postoperative dislocation. The CPP approach has a narrow surgical field compared to the conventional posterior approach. During surgery, the CPP approach needs extra care not to damage the muscle and retain it, especially the internal obturator tendon and inferior gemellus muscles. Therefore, this study was performed (1) to clarify the factors associated with internal obturator tendon and inferior gemellus muscle damage and (2) evaluate whether the muscle damages affect the postoperative dislocation rate in the CPP approach in BHA for femoral neck fractures in our institute.

## 2. Materials and Methods

### 2.1. Patients

This study was a single-center, retrospective, cross-sectional study approved by our hospital’s Ethics Committee. Informed consent was obtained in the form of an opt-out on the website. This study reviewed 185 consecutive hips from 183 patients with femoral neck fractures at our institution between April 2018 and November 2021. This study included patients with displaced femoral neck fractures who sought surgical treatment who were more than 65 years old. Patients were excluded if (1) surgery was deemed unfeasible due to poor general health, (2) they preferred conservative treatment, or (3) they were followed up for less than 6 months. In total, this study comprised 170 hips from 168 patients. All patients underwent unilateral primary BHA using the CPP approach. Of these, 142 hips were operated on by a senior surgeon, while 28 hips were operated on by fellows under the supervision of a senior surgeon. The preoperative prevalence of dementia among the patients was 37%.

Table 1 shows the baseline demographic data, including age, sex, follow-up periods, body mass index, and Garden classification [17]. The femoral components were applied for these patients depending on the surgeon’s opinion; in 161 hips, Type 1 cementless stems were used, such as Taperloc (Zimmer Biomet, Warsaw, IN, USA) and POLAR stem cementless (Smith and Nephew, Memphis, TN, USA) [18]. In 6 hips, Type 3C cementless stems were used, and in 3 hips, cemented stems were used [18].

### 2.2. Surgical Technique and Evaluating Muscle Damages

**Figure 1:**
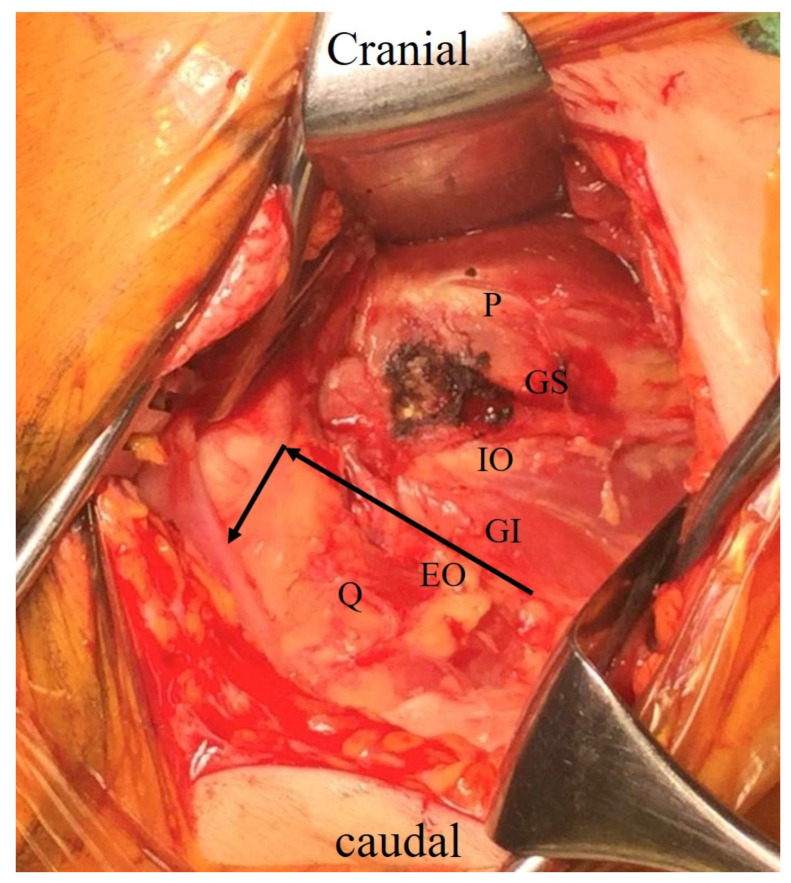
Exposed short eternal rotators. The black indicates the L-shaped resection line of the CPP approach. P: piriformis tendon.; GS: gemellus superior muscle; IO: internal obturator muscle; GI: gemellus inferior muscle; EO: external obturator muscle; Q: quadratus femoris muscle; CPP: conjoined tendon-preserving posterior.

The procedure of the CPP surgical procedure was similar to the previous reports [15,16]. BHA surgeries were performed by two well-trained orthopedic surgeons with more than 10 years of hip surgery experience. The procedure was carried out with the lateral position of the decubitus, with the affected side up. A straight skin incision of approximately 10 cm was made from the center of the vastus ridge in a proximal posterior direction with the hip in a 60° flex position. The gluteus maximus is separated bluntly along the muscle fibers to expose the greater trochanter, and the posterior boundary of the gluteus medius and the short external rotator muscles are detected. The piriformis tendon, gemellus superior, internal obturator, gemellus inferior, and external obturator muscles are identified. The capsule is incised along the caudal margin of the gemellus inferior muscle from the posterior border of the acetabulum to the posterior border of the femur (Figure 1). The incision is extended distally along the femur, and the external obturator muscle and the proximal two-thirds of quadratus femoris are included. This capsule is inverted, like the L-shaped flap (Figure 1). Then, the fracture site was developed.

**Figure 2:**
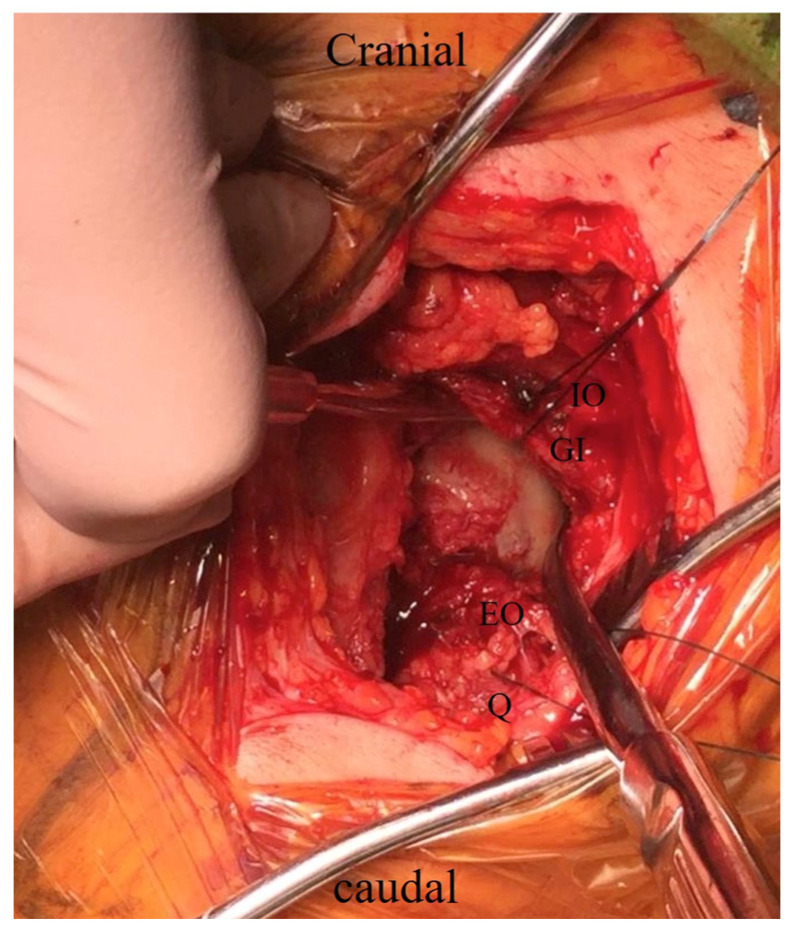
The femoral head was removed from the acetabulum using two elevators. IO: internal obturator muscle; GI: gemellus inferior muscle; EO: external obturator muscle; Q: quad-ratus femoris muscle.

**Figure 3:**
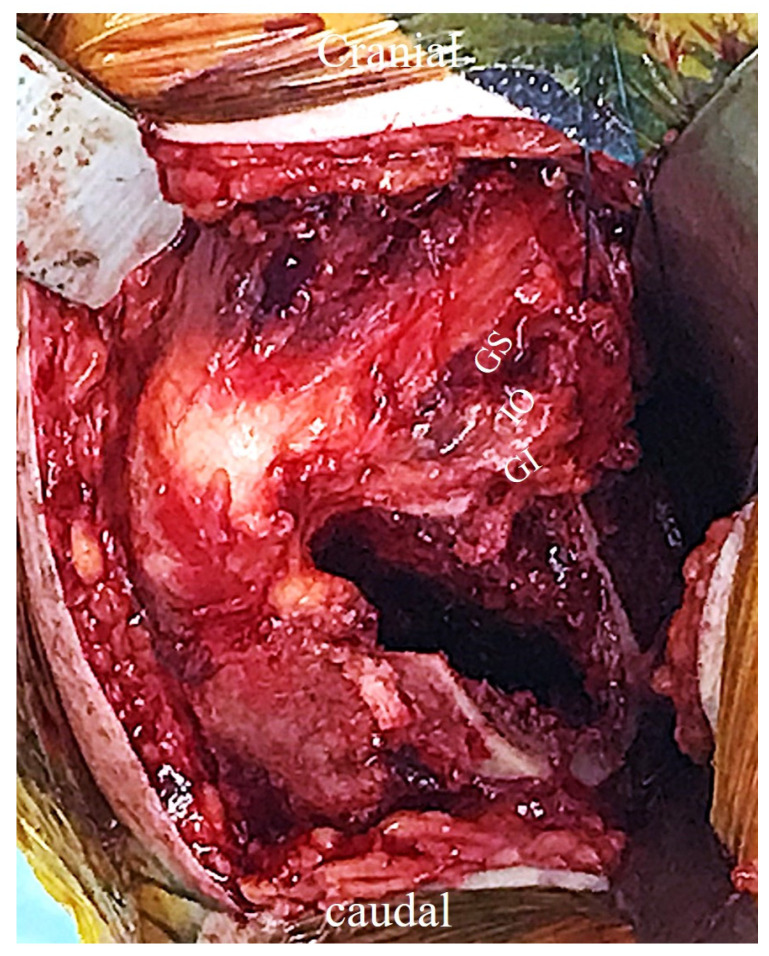
Femoral broaching is performed carefully so as not to damage the short external rotators. GS: gemellus superior muscle; IO: internal obturator muscle; GI: gemellus inferior muscle.

The femoral neck was cut along the planned osteotomy line, and the femoral head was lifted from the acetabulum using two elevators and then removed (Figure 2). The femoral head size was measured, and a trial cup was inserted into the acetabulum to determine the size of the outer head. The hip joint was flexed, adducted, and internally rotated to manipulate the femur in a similar way to the conventional posterior approach. Femoral broaching was performed carefully so as not to damage the short external rotators (Figure 3). The bipolar head reduction to the acetabulum was performed by pushing the outer head with the hip joint flexed approximately 90°. Minor adjustments were made intraoperatively. The same side implant was inserted.

The preservation of the short external rotator muscles was evaluated by direct visual inspection; the gemellus inferior and internal obturator tendons were damaged or not. The obturator internus muscle can be distinguished as its tendon components can be identified by two senior surgeons, N.A. and H.T. The external obturator muscle, quadratus femoris muscle, and capsule are repaired, and the incised quadratus femoris is repaired to the posterior border of the femur by interosseus suture, and then the wound is closed. In postoperative rehabilitation, patients were allowed a wheelchair and full weight bearing with a walker or crutches. The hip joint was flexed, adducted, and internally rotated to manipulate the femur in a similar way to the conventional posterior approach. No hip precautions were taken with the prescription of postoperative equipment and restrictions to functional activities. Conventional anteroposterior pelvis radiographs, including both hips, were obtained to check the implant fixation.

### 2.3. Clinical Evaluation

The duration of surgery and the amount of blood loss during the operation were recorded. The damaged short rotators, such as the gemellus inferior muscle and internal obturator tendon, were noted. Postoperative complications, including dislocation, infection, and neural palsy, were examined. The factors related to intraoperative short rotator muscle injury were investigated.

### 2.4. Statistical Analysis

Statistical analyses were performed using the EZR 1.41 (Saitama Medical Center, Jichi Medical University, Saitama, Japan) system, which is a graphical user interface for R 2.6-1 (The R Foundation for Statistical Computing, Vienna, Austria) [19]. A comparison of continuous variables was performed with the Mann–Whitney U test. The Kaplan–Meier product-limit method estimated the cumulative probabilities of life prognosis. Fisher’s exact test compared the muscle damage and femoral component type. All the statistical tests were two-sided, and *p*-values of <0.05 were considered statistically significant.

## 3. Results

Table 2 shows the result of the duration of surgery, amount of blood loss, damaged muscles, and postoperative complications. Four hips (2.3%) had damage to the obturator internus, and thirty-eight hips (22.4%) partially damaged the gemellus inferior muscle during the operation. A postoperative posterior dislocation occurred in one hip (0.6%), and a postoperative infection occurred in one hip (0.6%). The dislocated case was a female without damage to the obturator internus and gemellus inferior muscle. Peroneal or sciatic nerve paralysis was suspected in two hips (1.1%), one hip (0.6%) recovered within 3 weeks, and one (0.6%) hip remained paralyzed.

Table 3 indicates the factors related to intraoperative gemellus inferior muscle injury. Body mass index was the most relevant factor. There are no significant differences in the duration of surgery, age, sex, operators, and stem types used.

Table 4 indicates the factors related to intraoperative obturator internus muscle injury over the gemellus inferior muscle. Body mass index was the most relevant factor, too. Here, there are no significant differences in the duration of surgery, age, sex, operator, and stem types used.

**Figure 4:**
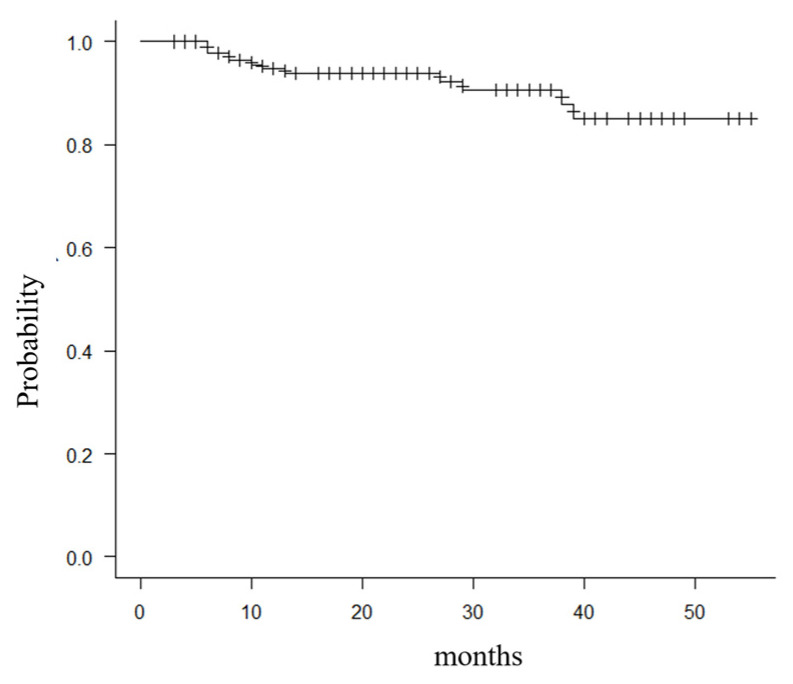
Kaplan–Meier survivorship analysis was 94.6% (95% CI 88.2–97.1) at 12 months after surgery, with deaths not related to fractures as the endpoint.

Kaplan–Meier survivorship analysis was 94.6% (95% CI 88.2–97.1) at 12 months after surgery, with deaths not related to fractures as the endpoint, and 87.6% (95% CI 77.5–93.4) at 36 months (Figure 4).

## 4. Discussion

In the present study, excellent postoperative results of the CPP approach in BHA for femoral neck fractures were achieved, considering the low postoperative dislocation rate and the high conservation rate of the obturator internus muscle despite the involvement of many geriatric patients. Delirium and dementia are risk factors for dislocation and are more prevalent in geriatric people [8]. The posterior wall consisting of the posterior capsule, the piriformis muscles, and the conjoined tendon composed of the superior gemellus, internal obturator, and inferior gemellus muscles were preserved. This `posterior wall` prevents postoperative dislocation and provides stability that addresses a wide range of motion of the hip. The deep external rotators have been proposed as critical active stabilizers of the hip, described as the ‘rotator cuff’ of the hip [20]; the CPP approach in BHA for femoral neck fractures may positively affect postoperative ADL recovery. The stability of hip joints to avoid dislocation increases the satisfaction of patients [21].

The most conventional posterior approach, the Moore or Southern approach, involves the conjoined tendon consisting of the short external rotators, the piriformis, the superior, obturator internus, and the inferior gemellus muscles release [22]. Furthermore, an obturator externus and a quadratus femoris were released as needed [22]. The dislocation rate with the posterior approach has been reported to be 3.8–13.0% [3,4,5,6,7,8]; the dislocation rate was lower with the repair of the joint capsule and external rotator muscles than without the repair [15]. However, once the capsule and short external rotator muscles are resected, the re-rupture rate is high (75–92%), even if being repaired [23,24]. Previous reports have described modifications to surgical procedures performed to reduce the incidence of postoperative dislocations after hemiarthroplasty or THA with a posterior approach [13,14,25,26]. The CPP approach in BHA for femoral neck fractures preserves the attachment of short external rotator muscles; the dislocation rate was reported as 0% [15,16]. There was one postoperative dislocation (0.6%) in the present study, and the dislocation rate was low compared to the previous reports of conversion posterior approach [3,4,5,6,7,8]. Improvement of daily living activities for patients and reduced caregiver burden was expected, especially for elderly patients. Further, the CPP approach enables early mobilization after surgery, which is expected to reduce DVT incidence and accelerate functional recovery. In one dislocated case, short external rotators, including the gemellus inferior, were retained during BHA. The short external rotators may be detached from their attachments, or the muscles may be torn when dislocated.

Although the CPP approach preserves the piriformis muscle from the gemellus inferior muscle, partial damage to the gemellus inferior muscle occurred. The previous study of the CPP approach reported that gemellus inferior muscle tear was detected in 4.4 to 10% [15,16], and the capsulotomy extended proximally from the caudal aspect of the inferior gemellus muscle was needed in 10% of cases [15]. The CPP approach has a narrow surgical field and technical demand compared to the conventional posterior approach. There may be cases in which the preservation of posterior soft tissues is difficult. There also may be a factor that the surgeons performing the CPP approach in this study included some residents and surgeons who were performing the approach for the first time [15,27]. In our data, the operator’s experience did not have much impact on muscle damage. The involvement of the senior surgeon in assisting the fellows might be beneficial. In our data, BMI was the most associated factor for muscle damage. Obesity may have affected the securing of the surgical field. Tetsunaga et al. reported that the Zweymuller-type stem was associated with inferior muscle damage to the gemellus [16]. A short taper stem was used in most cases, and there was no significant difference between the stem shape and gemellus inferior muscle damage. The 38 hips (22.4%) with partial gemellus inferior muscle damage had no postoperative dislocation, and the intact posterior capsule, ischiofemoral ligament, and internal obturator tendon seemed to be highly resistant to posterior dislocation [28]. The final condition of hips with partial gemellus inferior muscle damage was nearly equal to the condition of ERP, which preserves muscles from the piriformis to the internal obturator muscle [14]. The BHA with ERP has good outcomes with no cases of postoperative dislocation [14].

Postoperative neural palsy is a serious complication that has a negative impact on patients’ activities of daily living. In the present study, peroneal or sciatic nerve paralysis was suspected in two hips: one hip was recovered within 3 weeks, and one hip remained paralyzed. Peripheral nerve palsy following primary total hip arthroplasty is a relatively rare but potentially catastrophic complication [29]. Nerve palsies following total hip arthroplasty have been reported [29,30,31,32,33,34,35,36], and the prevalence of nerve palsy after total hip arthroplasty has been reported to range from 0.3% to 3.7% [29]. Another report identified 1.3% of primary arthroplasties as postoperative neuropathy of 3126 consecutive total hip replacements [33]. The sciatic nerve, or its peroneal division, was the most frequently injured, accounting for 79% of all nerve palsies [37]. The risk factors for nerve palsy were local trauma, including compression by intraoperative procedures [33,34], hematoma formation [35], compression secondary to cement protrusion [38], leg lengthening [30], female gender [35,37], and anticoagulant therapy [38]. Approximately 40% of patients will be asymptomatic over the course of 1 to 2 years; around 45% will be left with a slight deficit. Unfortunately, 15% are left with major motor and/or sensory deficits [37]. Although the cause of nerve damage in our study is not clear, it is possible the nerve was compressed or damaged during surgery. Since the surgical field is narrow in CPP, care must be taken to avoid compression of the sciatic nerve. Zoe et al. and Benoît Maeder et al. reported a rare case of sciatic nerve entanglement around a femoral prosthesis during the closed reduction in a dislocated total hip prosthesis [31,39]. Extra care must be taken to avoid engaging the nerve during intraoperative BHA reduction.

The present study’s survival rate was 94% at 12 months and 87.9% at 36 months after surgery, with deaths not related to fractures as the endpoint. Over the three years, the 1-year mortality rate for femoral neck fractures or trochanteric fractures in the patient population was reduced to 10.1% in our country [40]. A 1-year mortality rate after BHA for femoral neck fractures was 31.5%; key factors negatively influencing mortality at three months were cardiac complications, dementia, male sex, age, waiting time before operation, stroke, and dislocation of the prosthesis and perioperative fractures [41]. The postoperative mortality rate of the elderly with proximal femur fractures is about 10% [42]. A study focused on 367 proximal femur fracture patients who were ambulatory and living at home before the injury, and cases in which surgery was performed 2 days after hospitalization had a mortality rate of approximately 50% 1 year after surgery compared to cases in which surgery was performed after 3 days [43]. In our data, adverse events, such as death, did not occur in the perioperative period; the mortality rate is equivalent to past reports.

Our cohort study has several limitations that should be considered. Firstly, it is important to note that this study is a single-center retrospective study, which limits the generalizability of our findings. Further research in the form of a randomized multi-center study with a larger sample size would be beneficial to provide more robust results. Secondly, there is a limitation in the accuracy of assessing muscle damage. Our data rely on qualitative assessments of muscle damage, particularly regarding the gemellus inferior muscle. These assessments are based on direct visual inspection, which is qualitative in nature and not quantitative. The muscle damage should be evaluated ideally by two or more observers, not including the surgeons. Although we considered using CT scans for evaluation, it was challenging due to the presence of artifacts and proximity to the stem. Metal artifact reduction MR imaging has the potential to resolve this problem. The impact of muscle damage on functionality is also an area of interest that warrants further investigation. Lastly, a significant limitation is the absence of a control group of patients. However, using a conventional posterior approach, which is associated with a high risk of dislocation, in a comparative study could raise ethical concerns. Despite these limitations, our study provides valuable insights into the CPP approach’s effectiveness and its potential benefits for reducing postoperative dislocation in patients with femoral neck fractures.

## 5. Conclusions

The CPP method lowers the risk of postoperative dislocation when compared to the traditional posterior approach, making it an efficient option for performing BHA in cases of femoral neck fractures. It is worth noting that the obturator internus and the obturator internus damage do not have a significant impact on the occurrence of postoperative dislocation. However, it is important to acknowledge that individuals with a higher BMI are at a greater risk of experiencing partial damage to the gemellus inferior muscle. Overall, the CPP approach proves to be an effective choice for hemiarthroplasty in older patients with femoral neck fractures, especially those who may have risk factors for postoperative dislocation. However, caution should be taken for sciatic nerve palsy.

## Figures and Tables

**Table 1:** Patient demographic data.

Number of patients:hip joints	168:170
Age at the operation, mean ± SD (range)	81.2 ± 8.7 (65–101)
Gender, female:male, number of patients (%)	124 (73.8):44 (26.2)
Follow-up period, mean ± SD (range)	22.2 ± 15.9 (6–55)
BMI	20.4 ± 9.3 (13.4–30.2)
Garden classification	
I	0
II	5
III	68
IV	97
Stem selection	
Taperloc (Zimmer Biomet, Warsaw, IN, USA)	93
POLAR stem (Smith and Nephew, Memphis, TN, USA)	41
Anthology stem (Smith and Nephew, Memphis, TN, USA)	22
TriFit femoral stem (Corin, Tokyo, Japan; Cirencester, UK)	5
Alloclassic-SL (Zimmer Biomet, Warsaw, IN, USA)	3
SL-PLUS MIA (Smith and Nephew, Warsaw, IN, USA)	3
CPT hip system (Zimmer Biomet, Warsaw, IN, USA)	3

BMI: body mass index; data represent mean ± standard deviation.

**Table 2:** Surgical outcomes of the CPP approach.

Duration of surgery (min)	55.9 ± 15.0 (29–102)
Amount of blood loss (g)	124.9 ± 82.6 (35–475)
Muscle damage of short rotators	
Intact (%)	128 (75.3)
Gemellus inferior muscle (%)	38 (22.4)
Internal obturator tendon (%)	4 (2.3)
Postoperative complications	
Dislocation (%)	1 (0.6)
Infection (%)	1 (0.6)
Peroneal or sciatic nerve palsy (%)	2 (1.1)

Data represent mean ± standard deviation; CPP: conjoined tendon-preserving posterior.

**Table 3:** The factors related to gemellus inferior muscle injury.

	Non-Damaged	Damaged	*p*-Value
BMI	19.2 (13.4–27.8)	22.6 (16.1–30.2)	0.001 *
Duration of surgery	50.2 (29–99)	57.4 (35–102)	0.077
Age	83.6 (65–101)	81.4 (65–102)	0.701
Sex			
Female	93	31	
Male	32	12	0.683
Operator			
Senior surgeon	108	34	
Fellow	20	8	0.781
Stem selection			
Type 1	121	40	
Type 3C	4	2	
Cemented	1	2	0.232

Data represent the median (interquartile range); * *p* < 0.05; a Mann–Whitney U test was used to compare BMI, duration of surgery, and age. Fisher’s exact test was used to compare the sex and femoral component type.

**Table 4:** The factors related to obturator internus.

	Non-Damaged	Damaged	*p*-Value
BMI	20.4 (13.4–30.2)	23.5 (22.4–25.7)	0.047 *
Duration of surgery	53.6 (29–102)	67.8 (60–81)	0.067
Age	77.0 (65–102)	81.4 (70–101)	0.067
Sex			
Female	120	4	
Male	44	0	0.574
Operator			
Senior surgeon	139	3	
Fellow	27	1	0.526
Stem selection			
Type 1	159	2	
Type 3C	4	2	
Cemented	3	0	0.138

Data represent the median (interquartile range); * *p* < 0.05; a Mann–Whitney U test was performed to compare BMI, duration of surgery, and age. Fisher’s exact test was used to compare the sex and femoral component type.

## Data Availability

The data that support the findings of this study are available upon request from the corresponding author.
